# Editorial: Multilevel social determinants of individual and family well-being: national and international perspectives

**DOI:** 10.3389/fepid.2024.1381516

**Published:** 2024-03-26

**Authors:** Dillon T. Browne, Brae Anne McArthur, Nicole Racine

**Affiliations:** ^1^ Department of Psychology, University of Waterloo, Waterloo, ON, Canada; ^2^ Department of Psychology, University of Calgary, Calgary, AB, Canada; ^3^ Department of Psychology, University of Ottawa, Ottawa, ON, Canada

**Keywords:** family, social determinants, health, well-being, epidemiology

**Editorial on the Research Topic** Multilevel social determinants of individual and family well-being: national and international perspectives

For decades, the “social determinants of health perspective” has provided a framework around how the health of individuals is linked to social contexts, including family, school, community, neighborhood, peer, economic, political, and cultural phenomena. Increasingly, the family, itself, is being considered as a critical unit-of-analysis in understanding how social determinants shape life, health, and well-being. As this special issue attests, public health scholars have expanded upon individual health metrics to consider substantive processes within the family that have been historically prioritized by family therapists and psychologists (1). This exciting development is in the spirit of “multiple levels of analysis” (2, 3), championed in developmental psychopathology, whereby a unique and interdisciplinary mode of understanding emerges only when considering constructs that have historically resided in disciplinary silos. The nine papers in this special issue follow this theoretical spirit. Below, we have highlighted our key learnings.

1. *Incorporation of historical epochs into theoretical paradigms, including the pandemic, remains essential in understanding the impact of social determinants on individuals and families.*

Much of the present research utilized data sets that were mobilized during the pandemic. For example, using an impressive nation-wide survey quickly mobilized by Statistics Canada in the wake of the COVID-19 pandemic, Colucci et al. demonstrated that parents who had lower levels of education, experienced unemployment, or were essential workers had greater fears about child and family welfare during the pandemic. Using the same dataset, Zhang et al. demonstrated that families with higher socioeconomic status (SES) tended to have children with less media-saturated experiences during the shutdown and were more likely to plan on utilizing out-of-home childcare upon the pandemic's recession. Outside the pandemic, both Toombs et al. and Hicks et al. positioned their important contributions in the Truth and Reconciliation conversation in Canada, as outlined below. As ongoing global events continue to shape health and well-being for individuals and families, it is essential to incorporate these perspectives into research and policy.

2. *Health disparities must be articulated, while promoting non-pathologizing strengths-based perspectives that identify the undeniable resilience of people and their kin.*

Toombs et al. and Hicks et al. offer exemplary, empirical perspectives from the Aboriginal Peoples Survey in Canada (2017), demonstrating how racist, genocidal national policies (i.e., the Canadian Residential School System) have informed health for generations. Yet, they acknowledge these historical injustices while offering a strengths-based understanding, articulating the dignity of persons, and make policy recommendations that are culturally sensitive, informed, and consider the complexity of social determinants among First Nations Canadians. In a completely different context, Jia et al. similarly demonstrate the health consequences of historical harms perpetuated by the state (i.e., the Hukuo System in China), which are presently being addressed through policies aimed at reparation and healing. Furthermore, Toombs et al., Hicks et al. and Jia et al. demonstrate the power of articulating these historical health events, and their sequelae, from an empirical perspective using sophisticated epidemiological paradigms.

3. *Social determinants are not only important to consider for individual health, but also in relation to general family well-being.*

Social determinants in relation to family health and well-being is a recurring theme across most studies in this special issue. This is evident in contributions even when a traditional “family” outcome is not, necessarily, at the forefront of the research question. For example, Herrin et al. consider childhood wheezing and asthma from the lens of prenatal programming within families. While their initial hypotheses were not directly supported, the paradigm speaks to the importance of considering intergenerational exposure to health pathogens, which may further interact with biological sex and social contexts, demonstrating complexity in mechanisms of transmission. Similarly, Sivashankar and Chen consider the highly familial problem of substance use disorder during the pandemic, which importantly interacted with shame, social relations, and socioeconomic related variables (e.g., employment), identifying important differences across male and female respondents. This work is an extremely important direction, especially considering the massive rise in substance use problems globally, particularly for males (4).

4. *Public attitudes, including stigma and racism, continue to be barriers that challenge efforts to promote the health of individuals and families, while redressing historical harms.*

The relationship between public attitudes and stigma related to mental illness is noted in several abstracts. Pybus et al. investigated the relationship between national socioeconomic conditions and public attitudes regarding individuals with mental illness, underscoring the importance of reducing stigma at national levels. Furthermore, this impressive contribution denotes the multiple levels of analysis perspective underscoring the entire special issue. That is, both macro (i.e., gross domestic product and income inequality) and micro (i.e., difficulty paying bills) processes corresponded to stigma among the Eurobarometer sample (over 20 countries). From stigma to racism, Toombs et al. and Hicks et al. contextualize their important findings within the institutional racism that has plagued Canada for generations and is epitomized by the Residential School System. While acknowledging the complex, multilevel, and historical challenges inherent in reconciliation, they provide specific recommendations for grassroots, community interventions that can support mental health challenges in Indigenous families.

5. *Policy and intervention implications must continue to incorporate perspectives of family well-being, given the clustering of social determinants amongst related and co-residing persons and, consequently, health outcomes.*

While the papers in this issue are distinct, each highlighting specific issues related to social determinants of health in different health domains, geographies, and historical contexts, they overlap in the call for health policy and interventions that address the complex ecology of family life. Interventions cannot be uncoupled from social determinants of health and must simultaneously consider the cultural realities of families they are intended to reach. Anti-racist practices that acknowledge the historical harms of states and political institutions are indispensable in this effort. Moreover, the uptake and sustainability of health-promotion initiatives depends, in part, on broader socio-political conversations. This is, perhaps, an upsetting truism considering suggestions of a rise in global populism and extremism (5). Nevertheless, based on this collection of papers, the development, implementation, and evaluation of culturally sensitive, specific, and measurable intervention practices and policies—ones that acknowledge social determinants of health for individuals and families, within historical and current political contexts—is undoubtedly on the pathway forward.
